# Regression Metamodel-Based Digital Twin for an Industrial Dynamic Crossflow Filtration Process

**DOI:** 10.3390/bioengineering11030212

**Published:** 2024-02-23

**Authors:** Matthias Heusel, Gunnar Grim, Joel Rauhut, Matthias Franzreb

**Affiliations:** 1 Karlsruhe Institute of Technology (KIT), Institute of Functional Interfaces, Hermann-von-Helmholtz-Platz 1, 76344 Eggenstein-Leopoldshafen, Germany; matthias.heusel@kit.edu; 2 Andritz Separation GmbH, Industriestraße 1-3, 85256 Vierkirchen, Germany; gunnar.grim@andritz.com (G.G.); joel.rauhut@andritz.com (J.R.)

**Keywords:** digital twin, hybrid model, metamodel, dynamic crossflow filtration, industry scale

## Abstract

Dynamic crossflow filtration (DCF) is the state-of-the-art technology for solid–liquid separation from viscous and sensitive feed streams in the food and biopharma industry. Up to now, the potential of industrial processes is often not fully exploited, because fixed recipes are usually applied to run the processes. In order to take the varying properties of biological feed materials into account, we aim to develop a digital twin of an industrial brownfield DCF plant, allowing to optimize setpoint decisions in almost real time. The core of the digital twin is a mechanistic–empirical process model combining fundamental filtration laws with process expert knowledge. The effect of variation in the selected process and model parameters on plant productivity has been assessed using a model-based design-of-experiments approach, and a regression metamodel has been trained with the data. A cyclic program that bidirectionally communicates with the DCF asset serves as frame of the digital twin. It monitors the process dynamics membrane torque and transmembrane pressure and feeds back the optimum permeate flow rate setpoint to the physical asset in almost real-time during process runs. We considered a total of 24 industrial production batches from the filtration of grape juice from the years 2022 and 2023 in the study. After implementation of the digital twin on site, the campaign mean productivity increased by 15% over the course of the year 2023. The presented digital twin framework is a simple example how an industrial established process can be controlled by a hybrid model-based algorithm. With a digital process dynamics model at hand, the presented metamodel optimization approach can be easily transferred to other (bio)chemical processes.

## 1. Introduction

Since the term Industry 4.0 was introduced at the Hanover Fair in 2011, the importance of the development of cyberphysical production systems (CPPSs) is growing faster than ever in the biomanufacturing industry. CPPSs are physical production plants equipped with computation processing units used to automatically control the process. The realization of a cyberphysical system for manufacturing is generally understood to require, among other things, a virtual replica of the process that bidirectionally interacts with the physical process, the collection and usage of process data, and the optimization of the process with the help of intelligent algorithms [1]. The virtual part of a CPPS, including the digital process model and the algorithms, is called a digital twin [2,3].

Concepts and requirements for digital twins in manufacturing have frequently been presented and reviewed in the literature [4,5,6,7,8,9,10,11,12,13,14,15], whereas practical use cases where a digital twin has been developed for real-life processes are few to be found in the literature. Lopez and coworkers built a hybrid model for a fermentation process that is updated with data during the process [16], but they did not close the information loop from the digital part back to the physical process. A digital process replica with such an “one-way” information flow from the physical to the digital part is called a digital shadow [4,16]. Trunzer et al. used data-driven models trained with expert knowledge to identify the operational states of an industrial chemical reaction plant from the process data [17]. However, the models were not used for plant process optimization. Chew et al. used data-driven models to control a filtration process [18]. The optimization algorithm was tested on a laboratory filtration unit and a benefit for control of industrial filtration units has been predicted.

Digital twins rely on digital process models by definition, which are the backbone that makes process monitoring, simulation, and optimization possible [19,20,21]. Regarding the literature, mathematical models used to describe bioprocesses are divided into mechanistic and data-driven models [19,22]. Mechanistic models include models based on first principles, namely mass and energy balances and previous process knowledge. Data-driven models are built up on process data without incorporated previous knowledge. The trend in recent years is to combine mechanistic and data-driven models to create hybrid model structures in order to profit from the extrapolation capability of mechanistic models and the flexibility of data-driven models at the same time [22,23]. Mechanistic and data-driven models can be arranged in different serial or parallel configurations to hybrid models, depending on which modeling goal is to be aimed for [24,25]. The most commonly used model configuration in biochemical engineering is a serial structure of a data-driven model and a mechanistic model, wherein the data-driven model functions as a filler for overall model parts that cannot be expressed based on first principles [24].

Regarding the model description of the filtration processes, a spectrum of different approaches and use cases is presented in the literature [26]. Krippl and coworkers connected a neural network with mass balances in a serial model configuration to predict the permeate flux over time in a conventional crossflow filtration unit [27]. Piron et al. applied the serial model configuration to yeast filtration [28]. Díaz et al. used a serial neural network and Darcy law configuration for flux prediction, but did not use the model for process control and optimization [29]. Chew et al. used a similar model structure for the prediction of the fouling and filtration resistances of an industrial water clarification process; however, the model was not yet further used for process optimization [30].

A special type of model interconnection is the concept of metamodels or surrogate models [19,22,31,32]. A surrogate model is defined as a superordinated, simplified model based on the knowledge of the original, underlying model. The metamodel reduces complexity and increases calculation time to enable model calling during the real-time optimization of a process. Franzreb et al. used a model-based design-of-experiments (DoE) approach to make statements about the economic variables of a complex antibody production process [33]. Wang et al. trained a neural network with data generated from a mechanistic model of protein chromatography, and they recognized the advantages of the metamodel to quickly predict model parameters to adapt the mechanistic model to new feed streams [34]. Reports on a metamodel built for a filtration use case could not be found in the literature.

This paper is the first to present a digital twin for the process of dynamic crossflow filtration (DCF). We introduce a very quick regression metamodel to enable providing setpoint recommendations in almost real time. Furthermore, the practical implementation of the digital twin in an industrial use case is reported. DCF is a high-performance filtration technique that relies on rotating discs reducing the deposition of fouling components on a membrane surface [35,36]. The modeling of rotating membrane discs requires consideration of the physical phenomena occuring only with DCF such as cake reduction and fluid backpressure due to rotation of the membranes [37,38]. In this publication, the reader is shown how we aim at creating a digital twin of a brownfield DCF plant in order to enable optimized setpoint decisions in real time and to utilize the digital twin during an industrial filtration campaign, with the goal of improving the mean campaign DCF process productivity. Therefore, we built a mechanistic, semiempiric, hybrid process model of membrane fouling during the DCF of grape must. We present a concept of training a data-driven metamodel banking on model-based DoEs and response surface modeling and showcase how to straightforwardly apply the resulting metamodel for the time-critical forecasting of optimum process productivity. The model collective is implemented into a digital twin framework that reads the torque and transmembrane pressure (TMP) parameter at regular time intervals from the physical DCF plant during the process run and returns the permeate flow rate setpoint calculated for optimum productivity to the process actuator. The added value of introducing the digital twin to the industrial process is examined by comparing the overall productivity between 2022 and 2023 grape must filtration campaigns.

## 2. Materials and Methods

In this section, the plant setup of the industrial filtration runs and an examplary set of process data that is applied for model construction are presented. The structure and the elements of the digital model collective are explained, followed by a description of the superstructure of the digital twin framework.

### 2.1. Production Setup and Operation

**Figure 1:**
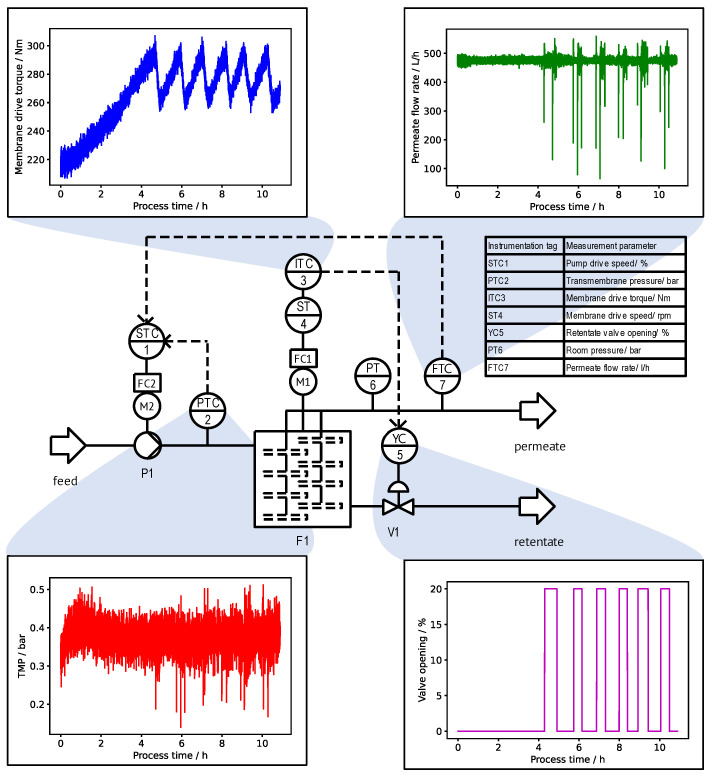
Piping and instrumentation diagram of the filtration process complemented by typical trends of central process variables during a filtration run. The plant setup includes sensors for TMP, permeate flow rate, membrane drive torque, and retentate valve opening. Actuators of the process control system are a feed pump, membrane drive, and retentate valve.

The experimental setup for the filtration runs is shown in Figure 1. The filtration machine (F1) is a DCF-type number 312/32 (Andritz Separation GmbH, Vierkirchen, Germany) equipped with four shafts and 256 ceramic membrane discs. Permeate passes through the porous membrane discs, and it is discharged from the machine inside the hollow shafts. Retentate is collected inside the process chamber and can be released by opening a valve (V1). The membrane discs are mounted on top of each other on every shaft, so that they form membrane stacks. Adjacent membrane disc stacks overlap, thus causing increased shear rate at the overlap region and achieving an increased cleaning effect from solid deposits at the overlapping points. The total available filter area is 32.8 m^2^; the membrane diameter is 312 mm.

The feedstock considered for the filtration experiments of this research study is pressed grapes containing pulp and other solids. Thus, the valuable product of the process is the clarified sweet must, i.e., the permeate. A membrane pore size of 200 nm is used for the filtration process. The filtration runs were performed at a production facility of a wine manufacturer in Italy during the regular sweet must production campaigns from August to October 2022 and 2023.

A centrifugal pump (P1) transports the feed from a storage tank into the DCF chamber. The feedstock is periodically mixed in the storage tank; however, a fully uniform solids content in the feed during the entire filtration process cannot be ensured. The pumping rate of the feed pump is controlled by a programmable logic controller (PLC) using the signal of sensors recording the TMP and permeate flow rate. The TMP is measured by the difference between the feed inlet pressure and the permeate outlet pressure, i.e., the TMP reduction due to membrane rotation is neglected. The filtration process starts in the “flow-controlled” operation mode; that is, the pumping rate of the feed pump is controlled at a constant permeate flow rate. The initial permeate flow setpoint is predefined by the plant operator at the beginning of the process run from experience dependent on estimated solids concentration in the feed and the postcleaning permeability of the membrane discs. As soon as the TMP setpoint of 0.8 bar is exceeded once during the production run, the operation mode automatically switches to “TMP-controlled”, and the feed pump rate is controlled to keep the TMP at a constant value.

The rotation speed of the membrane drive is controlled to a constant value of 343 min^−1^ during all experiments. The readout of power consumption from the frequency converter of the motor rotating the membrane shafts is done to receive data about the membrane drive torque. The membrane drive torque is strongly correlated to the retentate viscosity inside the DCF chamber and provides information about the increase in solids concentration in the retentate during a filtration run. Opening of the retentate valve for release of retentate from the DCF chamber is controlled by the membrane torque. At the beginning of the process, the DCF chamber is filled with feed suspension. During the concentration phase, the retentate valve is kept closed until the torque setpoint of 300 N m is reached. Subsequently, retentate discharge begins by gradual opening of the retentate valve until the torque does not increase any further. Closing of the retentate valve happens with a torque hysteresis of 10%, i.e., it closes only when the membrane torque falls below 270 N m.

The end of a filtration run is decided by the operator based on the amount of feed suspension available. A cleaning procedure with variable efficiency is followed by each filtration process. The permeate water flux is measured after every cleaning cycle for the assessment of the cleaning efficiency. All sensor and actuator setpoint data are logged 24/7 in 5 s intervals as a time series in a proprietary server database of Andritz (Metris All-in-One digitalization platform) and is used by us for model and digital twin validation after download as comma-separated values and manual segmentation into single filtration runs. When exporting data from the Metris platform, data preprocessing filters for, e.g., data compression by averaging are available; however, we have based our analysis on the raw data comprising approximately 2 MB for an experiment of 10 h in duration.

### 2.2. Mechanistic–Empirical Process Dynamics Model

A dynamics model that describes the physical process as close as possible is the backbone of the digital twin. We decided to build a mainly mechanistic model complemented with semiempirically chosen adjustment parameters in order to use existing process knowledge as a counterbalance to the limited variations in the available process data from filtration runs in industrial production.

**Figure 2:**
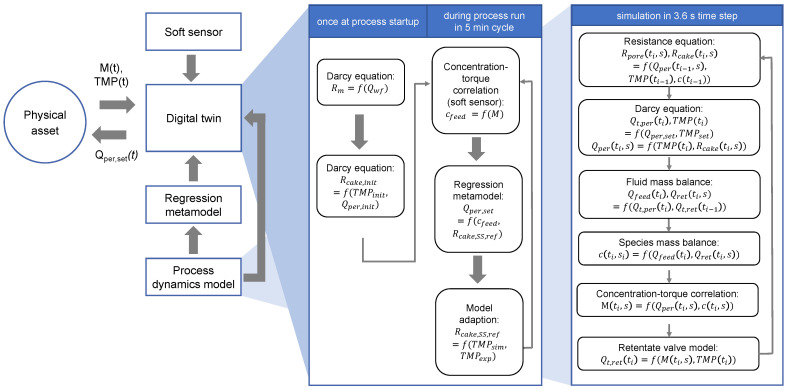
Overview of the digital twin structure. Obtaining torque data from the physical DCF asset, the digital twin applies a mechanistic–empirical soft sensor to estimate the feed concentration. The regression metamodel is used to find the optimum permeate flow rate setpoint, whereas the digital process dynamics model is called to assess the model validity in each process optimization cycle.

The calculation of the process parameters TMP, permeate, retentate and feed flow rates, membrane drive torque and valve opening percentage was implemented in a time loop with a cycle time of 3.6 s. In order to replicate the solids concentration gradient along the shaft axis from the feed inlet to the retentate outlet, the DCF chamber was conceptually divided into segments, and the permeate flow rate and membrane drive torque were calculated separately for each segment. The number of segments was set to $s_{\max}=$ 4 after a segment parameter study and a comparison of model simulation accuracy. The TMP was not calculated per segment, since the pressure drop along the shaft axis was assumed to be negligible. The sequence of calculation steps of the process dynamics model is presented in Figure 2 in the right-hand detail box.

The Darcy equation [39] was used to obtain the permeate flow rates during TMP-controlled operation mode or the TMP during flow-controlled mode, respectively:

(1) $$\mathrm{TMP}\left(t_{i}\right)=\frac{\eta \;\cdot \;Q_{\mathrm{per}}\left(t_{i},s\right)\;\cdot \;R_{\mathrm{tot}}\left(t_{i},s\right)}{A_{\mathrm{segm}}}$$

where η is the dynamic viscosity of water in Pa s, $Q_{\mathrm{per}}$ is the permeate flow rate in L h^−1^, $R_{\mathrm{tot}}$ is the total filtration resistance in m^−1^, and $A_{\mathrm{segm}}$ is the filtration surface area of one segment. The dependencies of the variables on time $t_{i}$ and the segment number *s* were marked. In the case of the flow-controlled operation mode, the calculation is not trivial, because although the total permeate flow rate is set by the operator or the digital twin, its distribution between the different segments of the DCF model is unknown at this stage of the time step. Therefore, in an intermediate calculation for each segment, a reference flow rate was calculated for a reference TMP. Subsequently, the sum of these reference permeate flow rates was compared to the desired total permeate flow rate, and the TMP was adjusted in order to match the flow rates. Knowing the required TMP, the real segmentwise permeate flow rates were calculated from the Darcy equation.

The resistance-in-series approach [39] was chosen for the modeling of the filtration resistances:

(2) $$R_{\mathrm{tot}}\left(t_{i},s\right)=R_{m}+R_{\mathrm{pore}}\left(t_{i},s\right)+R_{\mathrm{cake}}\left(t_{i},s\right)$$

where $R_{m}$ is the intrinsic membrane resistance in m, $R_{\mathrm{pore}}$ is the pore resistance in m^−1^, and $R_{\mathrm{cake}}$ is the cake resistance in m^−1^. The pore resistance was defined to follow an exponential relation:

(3) $$R_{\mathrm{pore}}\left(t_{i},s\right)=R_{\mathrm{pore},\mathrm{ref}}\;\cdot \;\left(\exp\left(k_{\mathrm{pore}}\;\cdot \;k_{p,i}\left(t_{i},s\right)\right)-1\right)$$

where $R_{\mathrm{pore},\mathrm{ref}}$ is the reference pore resistance in m^−1^, and $k_{\mathrm{pore}}$ is a model adaption parameter in L g^−1^m^−1^. The kinetic parameter $k_{p,i}\left(t_{i},s\right)$ is calculated as follows:

(4) $$\frac{dk_{p,i}\left(t_{i},s\right)}{dt_{i}}=c_{\mathrm{fine}}\;\cdot \;\frac{Q_{\mathrm{per}}\left(t_{i},s\right)}{A_{\mathrm{segm}}}$$

where $c_{\mathrm{fine}}$ is the concentration of fine particles in the DCF chamber in g L^−1^. Fine particles were assumed to pass through the membrane, and the fine particle concentration was set to 20% of the feed concentration. A rational equation of the Langmuir type [40] was used to model the cake resistance striving asymptotically to a maximum during filtration runs:

(5) $$R_{\mathrm{cake}}\left(t_{i},s\right)=R_{\mathrm{cake},\mathrm{SS}}\left(t_{i},s\right)\;\cdot \;\frac{k_{c,i}\left(t_{i},s\right)}{k_{c,i}\left(t_{i},s\right)+k_{\mathrm{cake}}}$$

where $R_{\mathrm{cake},\mathrm{SS}}\left(t_{i},s\right)$ is the steady state cake resistance in m^−1^, and $k_{\mathrm{cake}}$ is a model adaption parameter in g h L^−1^. The increase in the kinetic parameter $k_{c,i}\left(t_{i},s\right)$ in time is defined as being dependent on the chamber concentration:

(6) $$\frac{dk_{c,i}\left(t_{i},s\right)}{dt_{i}}=c\left(t_{i},s\right)$$

and the steady state cake resistance follows the equation:

(7) $$R_{\mathrm{cake},\mathrm{SS}}\left(t_{i},s\right)=R_{\mathrm{cake},\mathrm{SS},\mathrm{ref}}\;\cdot \;{\left(\frac{Q_{t,\mathrm{per}}\left(t_{i-1}\right)}{Q_{t,\mathrm{per},\mathrm{ref}}}\right)}^{n_{Q}}\;\cdot \;\exp\left(\frac{\mathrm{TMP}(t_{i-1})}{{\mathrm{TMP}}_{\mathrm{compress}}}\right)\;\cdot \;{\left(\frac{c(t_{i-1},s)}{c_{\mathrm{ref}}}\right)}^{n_{c}}$$

where $R_{\mathrm{cake},\mathrm{SS},\mathrm{ref}}$ is measured in m^−1^, $Q_{t,\mathrm{per},\mathrm{ref}}$ is measured in L h^−1^, ${\mathrm{TMP}}_{\mathrm{compress}}$ is measured in bar, $c_{\mathrm{ref}}$ is measured in g L^−1^, and dimensionless $n_{Q}$ and $n_{c}$ are model parameters. For process variable values that were taken from the previous time step of the simulation, the time variable was marked with the index i−1. The composition of the steady state resistance equation results from the analysis of the 2022 campaign’s process data and is based on physical knowledge about cake formation, cake shearing, and cake compressibility in filtration.

Fluid mass balances were implemented to obtain the retentate flow rates per segment, in which the feed flow rate of a segment is understood as the retentate flow rate of the previous segment:

(8) $$Q_{\mathrm{ret}}\left(t_{i},s\right)=Q_{\mathrm{ret}}\left(t_{i},s-1\right)-Q_{\mathrm{per}}\left(t_{i},s\right)$$

The overall feed flow rate of the process defines the inlet of the first segment:

(9) $$Q_{\mathrm{ret}}\left(t_{i},0\right)=Q_{\mathrm{feed}}$$

Chamber concentration values are calculated from segmentwise, instationary species mass balances:

(10) $$V_{\mathrm{segm}}\;\cdot \;\frac{dc(t_{i},s)}{dt_{i}}=Q_{\mathrm{ret}}\left(t_{i},s-1\right)\;\cdot \;c\left(t_{i},s-1\right)-Q_{\mathrm{ret}}\left(t_{i},s\right)\;\cdot \;c\left(t_{i},s\right)$$

with the following boundary condition:

(11) $$c\left(t_{i},0\right)=c_{\mathrm{feed}}$$

where *c* is the coarse solids concentration in the DCF chamber in g L^−1^, thus providing the concentration of particles and colloids retained by the membrane. The membrane drive torque is calculated from the solids concentration in two steps. Firstly, it is calculated via an empirical equation relating the viscosity exponentially to the concentration:

(12) $$\eta \left(t_{i},s\right)=\eta_{\mathrm{ref}}\;\cdot \;\exp\left(k_{\eta }\;\cdot \;f_{c}\left(t_{i}\right)\;\cdot \;c\left(t_{i}\right)\right)$$

where η is the dynamic viscosity in Pa s, $\eta_{\mathrm{ref}}$ and $k_{\eta }$ are model parameters, and $f_{c}\left(t_{i}\right)$ is an empirical correction function that takes into account that the suspended solids concentration in the DCF chamber does not exactly follow Equation (10), possibly because a fraction of the solids is fixed in the filter cake or attached to the chamber walls. In order to consider the effect, which results in a slowed down increase in the torque, $f_{c}\left(t_{i}\right)$ follows an exponential decay until the retentate valve is determined to open, thus indicating the approach of a quasisteady state in the DCF. Secondly, the well-known first principle mechanistic equation linking viscosity and torque [41] is applied:

(13) $$M\left(t_{i},s\right)=M_{\mathrm{seal}}+k_{g}\;\cdot \;\omega \;\cdot \;\eta \left(t_{i},s\right)$$

where $M_{\mathrm{seal}}$ is the torque already caused by the seals of the rotating shafts, and $k_{g}$ is the model parameter determined by the geometry of the DCF, mainly the total filter area and the distance between the discs.

The last calculation step is to obtain the total retentate flow rate from the valve opening:

(14) $$Q_{t,\mathrm{ret}}\left(t_{i}\right)=k_{\mathrm{valve}}\;\cdot \;\left(\mathrm{TMP}\left(t_{i}\right)\;\cdot \;X\left(t_{i}\right)-\sigma_{f}\right)$$

where $Q_{t,\mathrm{ret}}$ is the total retentate flow rate in L h^−1^, $k_{\mathrm{valve}}$ is the valve cross-section coefficient in L bar^−1^ h^−1^, *X* the valve opening in %, and $\sigma_{f}$ the flow stress of the retentate in bar. When the critical torque is reached, the degree of valve opening is gradually increased starting from a minimum opening percentage until the torque starts to descend.

For the simulation of the model, the initial permeate flow rate setpoint, the solid feed concentration, the process duration, and all model parameters have to be defined as input. After the digital process simulation, the mean productivity over a filtration run is obtained via a time averaging and space summation of the permeate flow rate:

(15) $$P=\frac{1}{t_{\mathrm{end}}}\;\cdot \;\int_{t=t_{0}}^{t_{\mathrm{end}}}\sum_{s=1}^{s_{\max}}Q_{\mathrm{per}}\left(t_{i},s\right)\;dt_{i}$$

where *P* is the average productivity of the production batch in L h^−1^, which we simply call productivity in the following, $t_{\mathrm{end}}$ is the process duration in h, and $s_{\max}$ is the dimensionless number of segments.

### 2.3. Regression Metamodel

A series of in silico experiments was performed with the process dynamics model to obtain the data for building the data-driven metamodel. A full factorial experimental design was chosen, and the initial permeate flow rate setpoint, the feed concentration, and the reference steady state cake resistance were varied in the boundaries of [400, 1200] L h^−1^, [20, 100] g L^−1^, and $\left[6\;\cdot \;10^{12},2.2\;\cdot \;10^{13}\right]\;m^{-1}$, respectively. Five levels were calculated per factor. All in silico experiments were carried out with a process duration of 10 h. Subsequently, a second-degree polynomial was fitted to the obtained values of the average productivities. Because of the strongly differing magnitudes of their numerical values, the factors had to be scaled to the interval [0, 1] before performing the fit. The calculations were performed using Python, including *pyDOE2*, as well as the *preprocessing.PolynomialFeatures* and *linear_model.LinearRegression* packages from the scikit-learn library.

Including the first- and second-order parameter terms, the interaction terms, and the constant bias term, the resulting response surface equation approximating the achievable productivity in dependence of the process parameters feed concentration, permeate flow rate setpoint, and steady state cake resistance has the form:

(16) $$\begin{array}{cc} P=w_{0} & +\;w_{1}c_{\mathrm{feed}}+w_{2}Q_{\mathrm{per},\mathrm{set}}+w_{3}R_{\mathrm{cake},\mathrm{SS}}+w_{4}{c_{\mathrm{feed}}}^{2}+w_{5}c_{\mathrm{feed}}Q_{\mathrm{per},\mathrm{set}}\\ \; & +w_{6}c_{\mathrm{feed}}R_{\mathrm{cake},\mathrm{SS}}+w_{7}{Q_{\mathrm{per},\mathrm{set}}}^{2}+w_{8}Q_{\mathrm{per},\mathrm{set}}R_{\mathrm{cake},\mathrm{SS}}+{w_{9}R_{\mathrm{cake},\mathrm{SS}}}^{2} \end{array}$$

where $w_{i}$ and i∈[0,9] are the parameters of the response surface that are identified via polynomial regression.

The aim of the digital twin is to optimize the productivity of the DCF. Therefore, the extrema of the predicted productivity of the metamodel are determined via the first-order derivative with respect to the permeate flow rate setpoint equaling zero:

(17) $$\frac{dP}{dQ_{\mathrm{per},\mathrm{set}}}=w_{2}+2w_{7}Q_{\mathrm{per},\mathrm{set}}+w_{5}c_{\mathrm{feed}}+w_{8}R_{\mathrm{cake},\mathrm{SS}}\overset{!}{=}0$$

The second-order derivative was checked to verify that the extremum found was a productivity maximum:

(18) $$\frac{d^{2}P}{d{Q_{\mathrm{per},\mathrm{set}}}^{2}}=2w_{7}\overset{!}{<}0$$

Equation (17) allows for real-time calculation of the permeate flow rate setpoint reaching for the maximum productivity of the process. Since the feed concentration is included, the optimum permeate flow rate setpoint can be easily recalculated when a change in the feed composition occurs during the filtration process run. If an unmodeled physical effect in the process causes a mismatch between the real and the modeled process dynamics, the reference steady state cake resistance parameter is used to adapt the mechanistic model during industrial DCF runs in 5 min intervals, if necessary. As well, since this parameter describing the physical properties of the solids in the feed is included as a factor in the response surface equation, changes in the natural feed solution sweet must during or between industrial runs do not require the repetition of the DoE prestudies.

### 2.4. Digital Twin Framework

The digital twin framework is built to use the model components and to communicate with the physical asset. The algorithm evaluates the plant status every 60 s by watching the PLC internal status variable. As soon as a process run is detected, the digital twin framework’s calculations are started. In Figure 2 the flow sheet of the digital twin framework is shown in the left-hand detail box. Once at the process startup, the intrinsic membrane resistance is calculated from the permeate water flux according to the Darcy Equation (1). Due to detectable cake formation already being present during the DCF chamber filling, the cake resistance parameter $k_{c,i}$ is initialized once at the moment that regular filtration starts using the measured TMP and permeate flow rate.

During the filtration process, the membrane drive torque is read every 5 min, and a permeate flow rate setpoint recommendation for the optimum productivity is returned to the DCF process control system. In every optimization cycle, the current feed concentration is estimated using the mechanistic–empirical correlation between the feed concentration and the torque from Equations (12) and (13) in the sense of a soft sensor. Secondly, the function based on a first-order derivative of the regression metamodel from Equation (17) is called, and the permeate flow rate recommendation is obtained for the current feed concentration and reference steady state cake resistance. Thirdly, the mechanistic–empirical model is used to simulate the past 5 min starting from the simulated status of the DCF obtained by the respective 5 min simulation in the previous optimization cycle of the digital twin. The simulation used the real experimental permeate flow rate setpoint and the current feed concentration. The resulting TMP from the simulation was compared to the actual TMP in the process. In the case where the TMP difference between the simulation and the experiment exceeded 0.1 bar, the reference steady state cake resistance was adapted by 10% for model refinement.

All program parts are written in Python and uploaded on the data server platform of the filtration plant. The platform serves as an interface between the digital twin framework and the local DCF process control system and allows for the reading of sensor data and writing of actuator instructions during the process. Due to safety reasons, changes in the permeate flow rate setpoints were manually authorized by the operator.

## 3. Results

The results section is divided into the presentation of the process dynamics model functions, the model validation with industrial experiment data, and the results of the metamodeling. The impact of applying the digital twin’s permeate flow rate recommendations on the 2023 process campaign is evaluated in the last section.

### 3.1. Mechanistic–Empirical Process Dynamics Model

**Figure 3:**
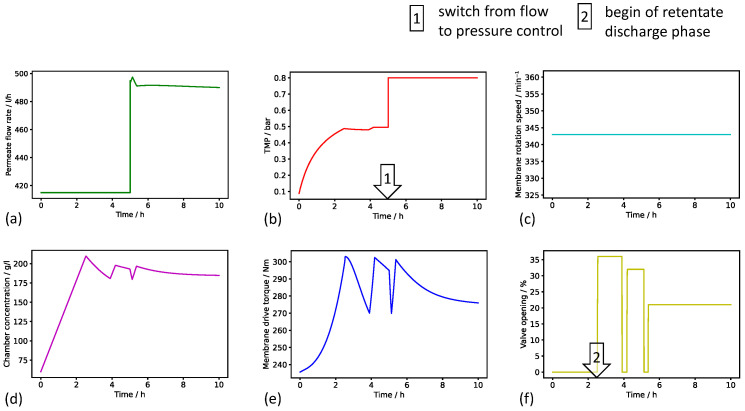
Simulation results of the digital process dynamics model for a 10 h in silico experiment. Time courses of (**a**) permeate flow rate, (**b**) TMP, (**c**) membrane rotation speed, (**d**) chamber solids concentration, (**e**) membrane torque, and (**f**) retentate valve opening are plotted. The permeate flow rate setpoint was defined to escalate after half of the simulated process time. The rotation speed was set constant. The modeled switch from flow to pressure control operation mode, and the beginning of the retentate discharge are marked.

A mechanistic–empirical model has been created to calculate the time course of all important process variables. Exemplary results of the predicted process dynamics can be seen in Figure 3. The simulation was defined to start with a permeate flow rate setpoint of 415 L h^−1^, and the target permeate flow rate was realized by the model system adjusting the TMP over time. The TMP increased during the initial 2.5 h of the modeled process, thus indicating a progressive membrane blocking. The slope of the TMP decreased during the initial 2.5 h of the modeled process, because the build up of the cake filtration resistance, that tended towards a maximum, was dominant over the increase in the pore filtration resistance. The process time 2.5 h after the start of the simulated process was characterized by the opening of the retentate valve; therefore, the TMP increase was stopped, and the TMP level was held. The membrane rotation speed was defined to remain constant. At the simulated process time of 5 h, the permeate flow rate setpoint was defined to increase by 80 L h^−1^ to showcase the switch from a flow-controlled to a TMP-controlled filtration mode. Until the end of the simulated process, the TMP was held at the predefined setpoint of 0.8 bar, and the permeate flow rate was calculated to slowly decrease because of the increasing pore filtration resistance.

Due to the constant permeate flow rate and a constant feed concentration of 60 g L^−1^, a linear increase in the lump chamber concentration calculated by the simple mass balance was observed until the retentate valve opened for the first time. Thereafter, the simulated concentration decreased and again increased in a manner dependent on the opening state of the valve. During the retentate discharge phase, the higher the valve opening percentage was, the higher retentate volume discharged and thus the faster the chamber concentration fell. The increase and decrease in the membrane drive torque correlated with those of the chamber concentration, thus following the modeled relationship between torque, viscosity, and concentration.

According to the results, the mechanistic–empirical model is capable of providing a simplified replication of the physical effects of membrane blocking and the control behavior of the filtration plant.

### 3.2. Process Dynamics Model Validation

**Figure 4:**
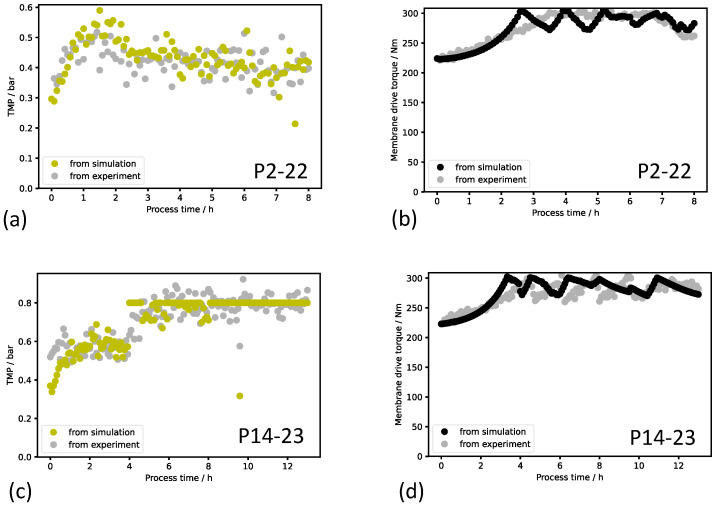
Validation of modeled TMP and torque with experimental data. The experiment from the 2022 campaign (TMP in (**a**) and torque in (**b**)) was carried out with a fixed permeate flow rate setpoint of 500 L h^−1^. In the the experiment from the 2023 campaign (TMP in (**c**) and torque in (**d**)), the permeate flow rate setpoint was increased from initial 400 L h^−1^ to 500 L h^−1^ after 4 h process time. The difference in the overall TMP level of both experiments can be explained by different membrane cleaning efficiency prior to the process run.

During the process runs with the digital twin, the current TMP and torque from the experiment and the model simulation were recorded after every optimization cycle. The plots resulting from two process runs are presented in Figure 4. A visual assessment shows a good agreement between simulation and experiment. The differences between the simulated and real TMP were smaller than the measurement noise. The increase in the TMP within the first hour of the process run 2–22 and the subsequent stabilization to a constant TMP level was recognized by the model. The initial, real TMP of run 2–22 was met by the simulation with a deviation of less than 10%. During the concentration phase of run 2–22, the increase in torque was adequately modeled for the initial 2 h of the process. However, the subsequent gradient of the torque increase was limitedly overestimated by the model such that the setpoint torque for the valve opening was reached 1 h earlier within the simulation. In run 2–22, the valve opening percentage was precisely set so that alternating opening and closing of the valve did not occur. This characteristic was properly simulated from the process run time of 5.5 h. Overall, the torque profile during the concentration phase and the retentate discharge phase was satisfactorily modeled.

Considering the TMP course validation of run 14–23, although the initial TMP was simulated to be 0.15 bar lower than the actual experimental value, the correction function of the digital twin adjusted the simulation within the first 30 min of the run. The shift in the TMP course at 4 h resulted from a change in the permeate flow rate setpoint from 400 L h^−1^ to 500 L h^−1^ and was reproduced by the model simulation. The constant TMP of 0.8 bar due to the change to the TMP-controlled operation mode at the time of the permeate flow rate setpoint change was simulated correctly. In the torque course of run 14–23, the increase during the concentration phase, the torque level during the retentate discharge phase, and the fluctuations due to the changes in the valve opening percentage were adequately approximated by the simulation.

All validation results of the 2022 and 2023 campaigns’ production runs can be viewed in Figures S1 and S2 in the Supplementary Information. During the filtration runs, the permeate flow rate setpoint was varied between 400 L h^−1^ and 850 L h^−1^ both between the experiments and partly during the single runs. Considering all the experimental validation results from the 2023 campaign, a good agreement between the model and real data has been reached particularly with natural variations in feed concentration and operational changes in the permeate flow rate setpoint, thereby underlining the robustness of the process dynamics model for this use case. The validated process dynamics model sets the basis for training the metamodel and for recommendations for optimum permeate flow rate setpoints of the digital twin.

### 3.3. Regression Metamodel

**Figure 5:**
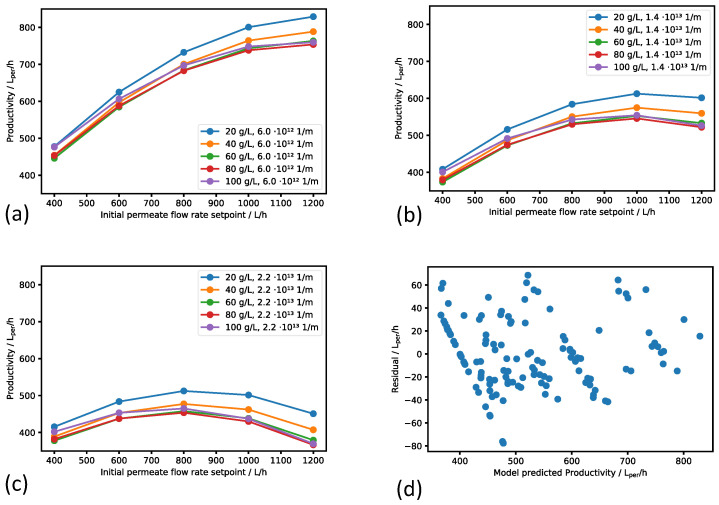
Results from the metamodel training. Subplots (**a**–**c**) show the dependency of productivity on initial permeate flow rate setpoint. Reference steady state cake resistance is constant in each plot, and feed concentration varies within the curve families. Subplot (**d**) presents the residuals of the regression.

A total of 125 in silico experiments were carried out with variation in the feed concentration, the initial permeate flow rate setpoint, and the reference steady state cake resistance. Using the productivity output from the in silico experiments, a regression metamodel was created, which allows for inverse calculation of the optimum initial permeate flow rate setpoint in the digital twin framework. The relationship between the initial permeate flow rate setpoint and the resulting experiment’s productivity in the metamodel is shown, together with the regression residuals, in Figure 5. With sparse exceptions, the absolute residuals from the polynomial regression were lower than 60 L_per_h^−1^, thereby indicating a satisfactory goodness of fit of the simple regression metamodel to the complex mechanistic model (cf. Figure 5d).

All in silico experiments with the initial permeate flow rate setpoint of 400 L h^−1^ resulted in a productivity of 400 L_per_h^−1^ within the accuracy of the regression model. With the initial permeate flow rate setpoint as low as 400 L h^−1^, the maximum TMP of 0.8 bar was not reached during the 10 h run, and, consequently, the permeate flow rate could be kept for the complete simulation. The same held true for the experiments having an initial permeate flow rate setpoint of 600 L h^−1^ and a reference steady state cake resistance of 6.0 · 10^12^ m^−1^. However, even in the case of this low cake resistance and the smallest feed concentration investigated of 20 g L^−1^, this 1:1 dependency between the productivity and initial permeate flow rate setpoint did not hold anymore when the initial permeate flow rate setpoint was increased to 800 L h^−1^. At the initial permeate flow rate of 800 L h^−1^, the maximum TMP of 0.8 bar was reached before the process time of 10 h was finished, and the process had to switch to the TMP-controlled operation mode. As a consequence, the permeate flow rate started to decay after this time point, and the (average) productivity of the run was less than the initially chosen permeate flow rate setpoint. In Figure 5a, the effect is shown in an increasing deviation of the course of the curves from a linear correlation of productivity with an increasing initial permeate flow rate setpoint. When the assumed reference steady state cake resistance was set to higher values of 1.4 · 10^13^ m^−1^ or 2.2 · 10^13^ m^−1^ (cf. Figure 5b,c, respectively), the time until the TMP setpoint was reached and the system switched to the TMP-controlled mode with reduced productivity became shorter. In addition, the compressibility of the filter cake started to show a significant negative effect on the productivity at higher cake resistances. The combination of these effects can result in a situation in which the selection of a too high initial permeate flow rate setpoint corresponds with a productivity that is lower than the productivity that could have been achieved if the process had been operated more carefully. Consequently, Figure 5b,c show the existence of an optimal initial permeate flow rate setpoint.

When the feed concentration increased, e.g., from 20 g L^−1^ to 40 g L^−1^, the productivity also decreased; however, the effect was minor compared to the effect of a change in the permeate flow rate setpoint or the reference steady state cake resistance on the productivity. The reason for the decrease in productivity can be found in the fact that higher solids concentrations accelerate the increase in filtration resistance over the process time. Consequently, the TMP increases faster, thereby resulting again in an earlier change from the flow-controlled to the TMP-controlled operation mode. In most cases, the influence of the feed concentration on the optimal initial permeate flow rate setpoint was quite small; however, at high reference steady state cake resistances, it can be seen that a feed concentration of 100 g L^−1^ shifted the optimum to lower values of the flow rate setpoint (cf. Figure 5c).

In summary, the variation in the optimum permeate flow rate setpoint with the model parameters reference steady state cake resistance and feed concentration confirms the relevance of a metamodel enabling the calculation of the appropriate permeate flow rate setpoint in the digital twin framework during the filtration process.

In the case of varying properties of the feed entering the DCF or an adjustment to the mechanistic model caused by an observed significant deviation in the simulated TMP from the experimental data, the metamodel makes it possible to conduct a new calculation of the optimal initial permeate flow rate setpoint in practically real time. The frequent calculation of the optimal initial permeate flow rate setpoint would not be possible when using the mechanistic model for optimization calculations directly. To find the optimum of the productivity resulting from a 10 h filtration, several runs of the mechanistic model covering the full process time would be needed, thereby resulting in a computation time clearly exceeding the 5 min intervals of the optimization loop of the digital twin.

### 3.4. Effect of the Digital Twin on the Productivity

**Figure 6:**
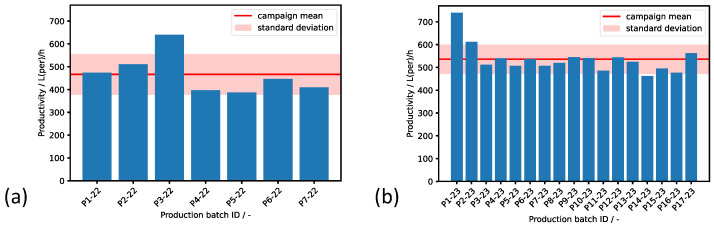
Productivity comparison between the production runs of the (**a**) 2022 and (**b**) 2023 campaigns. The digital twin was active from beginning of the 2023 filtration runs. A significant increase in average productivity of 15% was achieved from 2022 to 2023.

By the start of the 2023 production campaign, the digital twin was implemented on the server platform and put into operation. At this stage of the development, the permeate flow rate recommendations from the digital twin were transferred manually to the physical process by the operator during the course of the production runs due to safety reasons. The productivities of the 2022 and 2023 campaigns’ production runs and the campaign mean productivity are presented in Figure 6.

The average productivity of the productions runs in the 2022 campaign was $466\;L_{\mathrm{per}}h^{-1}$ ± $88\;L_{\mathrm{per}}h^{-1}$. An increase in average productivity of 15% was achieved from the 2022 campaign to the 2023 campaign, as the average productivity of the 2023 campaign was $536\;L_{\mathrm{per}}h^{-1}$ ± $63\;L_{\mathrm{per}}h^{-1}$. This shows that, in addition to the increased productivity, a reduction in the fluctuations of the productivity of individual runs by one quarter could be achieved by implementing the digital twin. We performed a classic *t* test for unpaired samples and reached a *p* value of 0.040. Accordingly, the increase in the 2023 campaigns’ productivity mean values can be classified as significant. The variations in the productivity of the individual runs during both filtration campaigns are explained by the different feed characteristics of the respective batches. For instance, the solids concentration, fouling particle compressibility, and temperature influence the degree of membrane blocking and thus the maximum applicable permeate flow rate setpoint.

The achieved increase in productivity in the campaign applying the digital twin can be mainly attributed to fact that the digital twin suggested initial permeate flow rate setpoints that resulted in a DCF operation at higher TMPs. In the manual operation during the 2022 campaign, the operators strived to avoid a transition into TMP-controlled operating conditions. However, the simulation runs and metamodel showed that the reference steady state cake resistance of sweet must was only moderate and that a transition into the TMP-controlled operation mode in the course of the filtration did not harm the productivity. These predictions were confirmed in the 2023 campaign, as can be seen in the individual TMP time courses of all runs’ plots in Figure S1 in the Supplementary Information.

## 4. Discussion

The results are discussed with regard to the quality of the mechanistic–empirical process dynamics model and the digital twin’s faculty to identify the optimum process setpoint.

### 4.1. Process Dynamics Model Quality

Generally, the mechanistic–empirical process model provides a good replication of the time dynamics of the process variables TMP and membrane drive torque. However, there were effects observed during some process runs that cannot be explained with current process knowledge. For instance, the initial TMP of process run 14–23 in Figure 4 was unexpectedly high, and the subsequent TMP course stayed constant within 4 h despite a constant permeate flow rate and a closed retentate valve. Nonetheless, the adaption functionality of the digital twin recognized the TMP deviations and corrected the model simulation. The metamodel was trained using the fundamental mechanistic–empirical model, i.e., the metamodel does not take the individually observed, unmodeled effects in the production data into account. A further development of the digital twin with regard to the ability to learn on the basis of observed effects in current or past production runs can possibly improve the model.

The development and iterative refinement of the process dynamics model was based on the data of the complete sweet must production campaign in 2022. However, the industrial production runs in 2022 were often carried out with similar setpoint values, thereby giving only limited variation to the process data. We tried to compensate for the limited information available from the data by considering the expert knowledge from project partners and process data obtained with other feed solution types. Nevertheless, the acquisition of data from further industrial filtration runs with variations in, e.g., the TMP maximum setpoint, permeate flow rate setpoint, and membrane drive torque setpoint, followed by the data integration into further model development would be desirable.

### 4.2. Digital Twin Optimization Capability

The capability of the digital twin to optimize the process not only depends on the quality of the process dynamics model, but it also depends on the accuracy of the trained metamodel. To generate the training data for the metamodel, in silico experiments were carried out, with the duration of all experiments fixed at 10 h. Originally, the real productions runs were also planned to be carried out with a fixed duration of 10 h to ensure precise comparability. However, in practice, the duration of the industrial runs is primarily determined by the amount of feed available at the time of the filtration run, and it is not set by the operator in advance. Thus, the duration of the filtration runs varies considerably over a campaign. Here, the optimization potential can be further exploited by matching the duration of the industrial run and the in silico experiment duration of the metamodel. For this, the expected duration of the industrial runs needs to be known. In the case of variable durations of the industrial runs, the duration can be added as an additional factor to the design space of the in silico experiments, thus extending the metamodel by one parameter.

More generally, the concept of the regression metamodel based on in silico process simulations offers the ability to integrate further operating, model, or geometry parameters of the underlying mechanistic–empirical process dynamics model into the metamodel. Therefore, only the in silico parameter study and the regression modeling need to be redone; experimental runs on site are not required. Possibly, besides the reference filter cake resistance, some more currently fixed model parameters could be changed to an adaptable form to improve the predictive power of the process dynamics model during the industrial runs. In addition, a higher flexibility of the modeled operation conditions, such as a permeate flow rate ramp, can be added to open new possibilities for productivity optimization. Lastly, the optimization objective is imagined to be extended from only productivity to other objective variables, such as the product yield, energy demand of the membrane drive, or membrane cleaning effort.

### 4.3. Applicability of the Digital Twin Concept beyond the Presented Use Case

The scalability of the digital twin concept in the scope of DCF systems, the transferability to other feed materials, and the applicability to other chemical processes are discussed below. Due to the mainly mechanistic nature of the model, the digital twin is expected to be applicable to other DCF systems with adjustments to geometry parameters such as the membrane diameter, the number of segments, or the geometry factor connecting the torque and concentration. The general principles based on the mechanistic model, such as cake formation, pore fouling, the influence of membrane rotation, and the PLC control mechanisms to maintain a constant TMP or permeate flow rate, remain consistent across all DCF systems. Extending the digital twin concept to other feed streams is considered feasible, assuming that the model parameters are tailored to the specific feed stream. However, the adaptability of the digital twin concept is currently constrained by the need to generate and evaluate experimental data in order to identify the model parameters. Looking ahead, incorporating an automated learning algorithm to facilitate model parameter identification during the process run would be advantageous.

The digital twin concept, with its polynomial response metamodel, is universal and appears to be transferable to other industrial processes. Nevertheless, the data-driven metamodel is reliable only within the design space, thus meaning it lacks extrapolation capability. Thus, the scope of the model parameters and operating conditions must be established during the training of the metamodel and can only be modified by retraining the metamodel. Moreover, the effectiveness of the metamodel depends on the availability of a robust process model. Therefore, the primary constraint on the universal applicability of the digital twin concept to other chemical processes likely lies in the need for a rigorous process dynamics model, which must be built on either detailed process knowledge or a comprehensive dataset that includes a significant range of variations in the system variables’ responses.

## 5. Conclusions

This study reports the development of a digital twin for an industrial DCF process and its successful application during the 2023 grape must filtration campaign resulting in an overall campaign productivity increased by 15%. The digital twin was constructed around a process dynamics model that we based on mechanistic filtration knowledge and fine-tuned with empirical correlations derived from historical process data. The validity of the mechanistic–empirical process dynamics model was confirmed by comparing torque and TMP time series with experimental data of process runs. A regression metamodel was interposed between the basal process dynamics model and the digital twin framework, because we found that the metamodel approach is superior to several calculations of the mechanistic–empirical process dynamics model in terms of computational speed and, together with the step-by-step simulation of the process dynamics model, and a setpoint recommendation was obtained within seconds. Examination of the regression residuals indicated a good fit between the simple metamodel and the underlying, complex mechanistic model. By comparing the TMP live data with the simulation result, the process dynamics model was continuously validated during the production runs. If a nonreplicated process effect occurs, the digital twin adjusts a model parameter included in the metamodel. As it could be seen in retrospect to the experiments, the metamodel approach held under deviations in the filtration system’s startup behavior, as well as in the case of changing filtration properties of the feed suspension; thus, the digital twin enables the robust prediction of an optimal permeate flow rate setpoint within a filtration process dealing with biological feed materials.

Given a mechanistic process model, the presented method can be used to optimize a (bio)chemical process without having to perform repeated computation-intensive calculations during the operation. The expandability of the metamodel offers the prospect of integrating further parameters into the optimization or optimizing additional target variables without additional experimental effort on site. Nonetheless, the transfer of the presented regression metamodel-based digital twin concept to other use cases is tied to the availability of a rigorous process dynamics model built up on either mechanistic process knowledge or a sufficiently large and variant amount of process data. 

## Data Availability

The data are not available due their proprietary nature with regard to the industry partner.

## Supplementary Materials

The following supporting information can be downloaded at: https://www.mdpi.com/article/10.3390/bioengineering11030212/s1, Figure S1: Overview of TMP courses of all production runs performed during 2022 and 2023 campaigns in comparison with the corresponding simulation results; Figure S2: Overview of torque courses of all production runs performed during 2022 and 2023 campaigns in comparison with the corresponding simulation results.
