# Supplementary material for: Regression Metamodel-Based Digital Twin for an Industrial Dynamic Crossflow Filtration Process
Source: Bioengineering (Basel). 2024 Feb 23;11(3):212. doi: 10.3390/bioengineering11030212 (PMC10967869; doi:10.3390/bioengineering11030212)
Supplement: Supplementary file 1 [file bioengineering-11-00212-s001.zip › bioengineering-2866376-supplementary.pdf]

Supplementary Information

as part of the article

Regression metamodel-based digital twin for an industrial dynamic crossflow filtration process

Matthias Heusel [1], Gunnar Grim [2], Joel Rauhut [2], Matthias Franzreb [1]*

[1] Karlsruhe Institute of Technology (KIT), Institute of Functional Interfaces, Hermann-von-Helmholtz-Platz 1, 76344 Eggenstein-Leopoldshafen, Germany
[2] Andritz Separation GmbH, Industriestraße 1-3, 85256 Vierkirchen, Germany
* Correspondence: matthias.franzreb@kit.edu

This document contains Figure S1 and Figure S2.

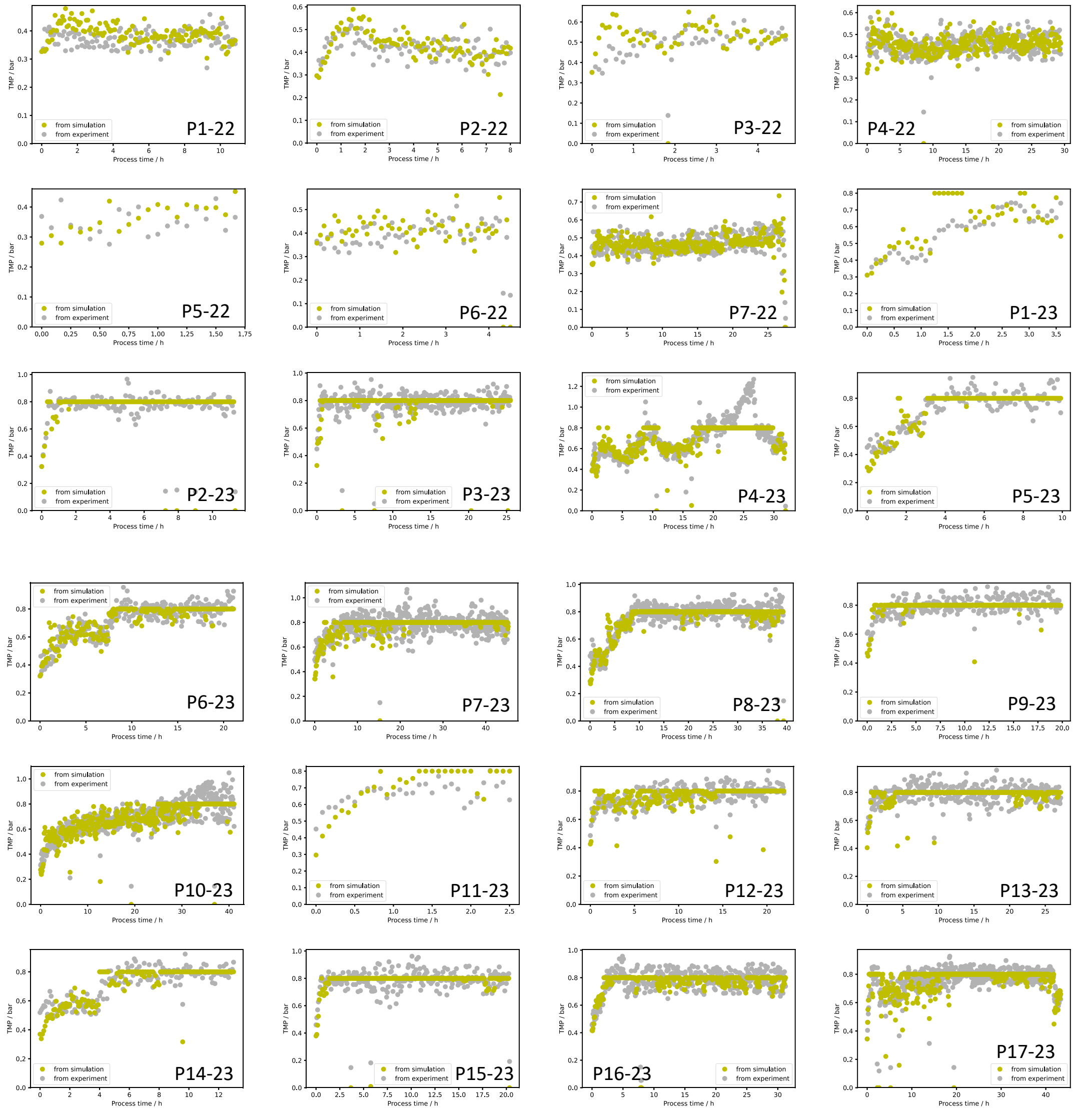

Figure S1: Overview of TMP courses of all production runs performed during 2022 and 2023 campaigns in comparison with the corresponding simulation results. TMP increases and the TMP maximum setpoints are well replicated by the digital twin in all process runs. However, if TMP is manually allowed to exceed the 0.8 bar setpoint (cf. P4–23), the simulation does not follow.

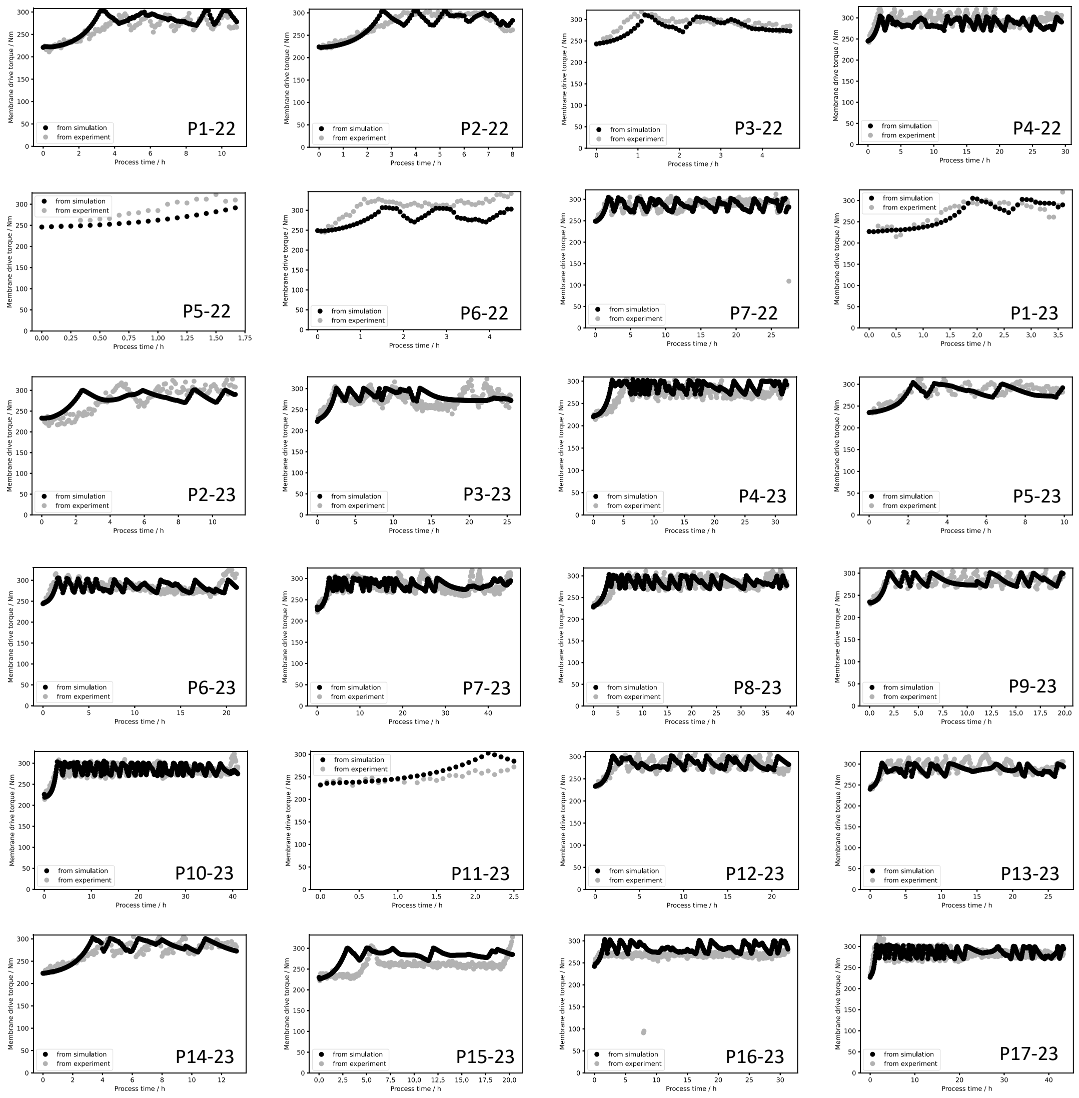

Figure S2: Overview of torque courses of all production runs performed during 2022 and 2023 campaigns in comparison with the corresponding simulation results. The digital twin replicates the torque curves adequately, although the frequency of the valve opening and closing cycles is not exactly matched.
